# GLP-1 receptor agonists in atherosclerotic cardiovascular disease and diabetes mellitus or obesity: investigation of the potential role in a German inpatient dataset

**DOI:** 10.1007/s00392-025-02735-z

**Published:** 2025-08-21

**Authors:** Vera Oettinger, Constantin von zur Mühlen, Klaus Kaier, Dennis Wolf, Jonathan Rilinger, Alexander Maier, Markus Jäckel, Dirk Westermann, Ingo Hilgendorf, Dalibor Bockelmann

**Affiliations:** 1 Department of Cardiology and Angiology, University Heart Center, Medical Center – University of Freiburg, Faculty of Medicine, University of Freiburg, Hugstetter Str. 55, 79106 Freiburg, Germany; 2 Center for Big Data Analysis in Cardiology (CeBAC), Department of Cardiology and Angiology, University Heart Center, Medical Center – University of Freiburg, Faculty of Medicine, University of Freiburg, Freiburg, Germany; 3 Institute of Medical Biometry and Statistics, Faculty of Medicine and Medical Center – University of Freiburg, Freiburg, Germany; 4 Medical Strategy Unit, Medical Center – University of Freiburg, Faculty of Medicine, University of Freiburg, Freiburg, Germany

**Keywords:** Atherosclerotic cardiovascular disease, ASCVD, Diabetes mellitus, Obesity, GLP-1 receptor agonist, Electronic health records

## Abstract

**Background:**

GLP-1 receptor agonists (GLP-1 RA) reduce major adverse cardiovascular events in patients with diabetes mellitus and atherosclerotic cardiovascular disease (ASCVD) or increased cardiovascular risk. Recently, the GLP-1 RA semaglutide was reported to reduce cardiovascular events in ASCVD patients with overweight or obesity even in the absence of diabetes mellitus. Accordingly, this was included in the current European Society of Cardiology guidelines for secondary prevention. We aimed to assess the number of inpatients with ASCVD who could qualify for therapy with GLP-1 RA.

**Methods:**

Using nationwide data from the Research Data Centre of the Federal Statistical Office of Germany, a random 10% sample of all adult inpatients in 2022 was analyzed. The proportions of ASCVD, obesity, and diabetes mellitus were examined.

**Results:**

The 10% sample included 1,446,420 patients with a mean age of 63.16 years, 52.50% were female. ASCVD was present in 229,619 inpatients (15.87%), including those with coronary artery disease, history of myocardial infarction, peripheral vascular disease or stroke. Among ASCVD patients, 79,516 individuals (34.63% of ASCVD patients) also suffered from type 2 diabetes mellitus and 15,624 individuals were diagnosed with obesity. Approximately half of those (*N* = 7,256) had ASCVD and obesity without diabetes mellitus (3.16% of ASCVD patients). Overall, 37.79% of ASCVD patients also suffered from type 2 diabetes mellitus or obesity.

**Conclusion:**

In this data sampling of German inpatients, more than one-third of ASCVD patients may qualify for GLP-1 RA therapy due to concomitant diabetes mellitus or obesity. That corresponds to about 6% of inpatients in Germany.

**Graphical Abstract:**

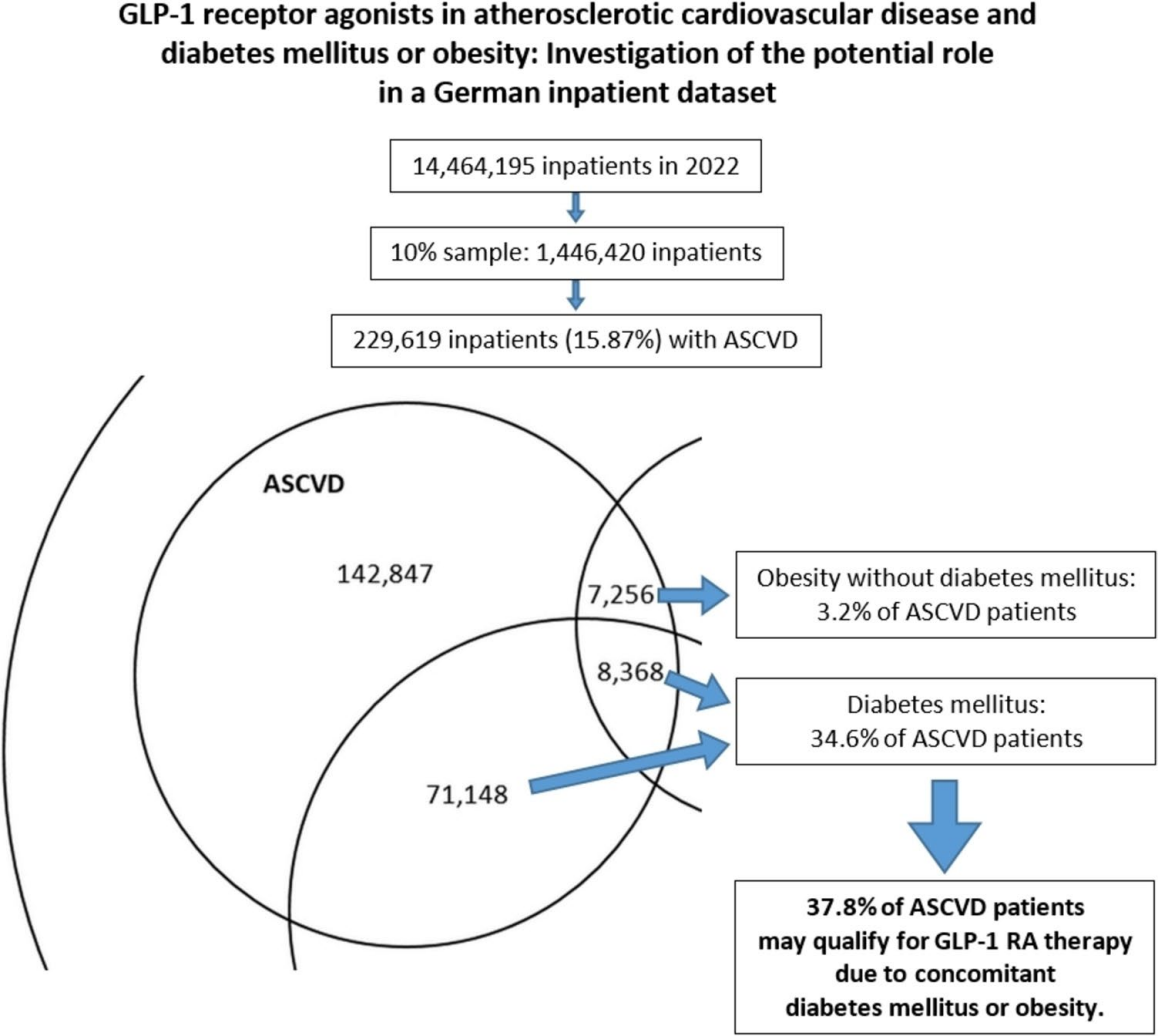

## Introduction

Diabetes mellitus [1, 2] and obesity [3–5] are well-established risk factors for the development as well as the progression of cardiovascular disease (CVD) and atherosclerotic cardiovascular disease (ASCVD). Diabetes mellitus is attributed a global prevalence of 10.5% [6] and obesity of 14.0–18.5% [7]. Although obesity can lead to the development of type 2 diabetes mellitus, dyslipidemia, as well as arterial hypertension [8], obesity alone, even in the absence of these metabolic disorders, can increase the risk for heart failure and ASCVD [9].

Glucagon-like peptide-1 receptor agonists (GLP-1 RA) [10–13] demonstrated cardiovascular benefits in patients with type 2 diabetes mellitus. Furthermore, GLP-1 RA such as semaglutide [14–18] and liraglutide [19–22] as well as the dual GLP-1/glucose-dependent insulinotropic peptide (GIP) receptor agonist tirzepatide [23–26] induce substantial weight loss in obese patients beyond glycemic control. In the recent SELECT trial [27], semaglutide showed a 20% reduction in risk of death from cardiovascular events, myocardial infarction, and stroke after treatment for 33 months in patients with CVD as well as a body mass index (BMI) ≥ 27kg/m^2^ even in the absence of diabetes mellitus.

Accordingly, current European Society of Cardiology (ESC) guidelines [28] for the management of patients with chronic coronary syndrome recommend GLP-1 RA for those with type 2 diabetes mellitus (class I, level A) and the GLP-1 RA semaglutide for those without diabetes mellitus when being overweight (BMI ≥ 27kg/m^2^) or obese (class IIa, level B). Likewise, the ESC guidelines [29] for the management of cardiovascular disease in patients with diabetes mellitus recommend the use in those with concomitant ASCVD. In this study we asked, how many ASCVD patients hospitalized in Germany would qualify for GLP-1 RA treatment according to these recommendations.

## Material and methods

The Research Data Centre of the Federal Statistical Office of Germany (Destatis) has been providing Diagnosis Related Groups (DRG) statistical data on all hospital admissions in Germany since 2005 for the purpose of scientific use. This dataset comprises nationwide inpatient treatment diagnoses as well as procedures of patients reimbursed under the DRG system. From this, all cases of patients aged 18 years and older who were hospitalized in Germany in 2022 were extracted, resulting in a total of 14,464,195 cases. For reasons of data economy, a random sample of 10% was drawn by applying Stata’s splitsample command, yielding 1,446,420 cases. The analyses were conducted using Stata 18, a software developed by StataCorp, located in College Station, Texas, USA.

The investigators in this study did not have direct access to individual patient data for this analysis. Access was granted by Destatis only for summarized results. Ethics committee approval and informed consent were therefore not required according to German law. Destatis anonymised all pooled results and any information that could potentially identify an individual patient or an individual center has been censored by Destatis to ensure privacy.

ASCVD, risk factors, and comorbidities were identified using ICD-10-GM codes, based on Lincoff et al [27]. The coding is as follows:

Codes I25.11, I25.12, and I25.13 encompass coronary artery disease, I25.20, I25.21, and I25.22 refer to history of myocardial infarction, I63*, I64, and I61 refer to history of ischemic or hemorrhagic stroke, as well as I70.2* and I70.9 refer to peripheral vascular disease.

Obesity is coded as E66*. The severity of obesity is being graded as:

- Obesity grade I (BMI = 30–34.9kg/m^2^): codes E66.00, E66.10, E66.20, E66.80, and E66.90
- Obesity grade II (BMI = 35–39.9kg/m^2^): codes E66.01, E66.11, E66.21, E66.81, and E66.91
- Obesity grade III (BMI ≥ 40kg/m^2^): codes E66.06, E66.16, E66.26, E66.86, E66.96, E66.07, E66.17, E66.27, E66.87, E66.97, E66.08, E66.18, E66.28, E66.88, and E66.98
- Unspecified level of obesity: obese patients who were not defined as grade I-III or whose grade was unknown

Code E11* refers to type 2 diabetes mellitus. Hypercholesterolemia is captured by E78.0. The New York Heart Association (NYHA) classes II-IV of heart failure are covered by the codes I50.12, I50.13, and I50.14. I10* refers to arterial hypertension. History of cardiac surgery is coded as Z95.1, Z95.2, Z95.3, and Z95.4. Carotid disease is being referred to by I65.2, chronic obstructive pulmonary disease (COPD) by J44*, as well as pulmonary hypertension by I27*. Renal disease is coded by N18.4 and N18.5 as well as atrial fibrillation by I48.0, I48.1, and I48.2.

Variables are presented as percentages or means with standard deviations. Categorical data were compared using the chi-squared test, while continuous data were compared using the unpaired t-test.

## Results

### Characteristics of patients hospitalized in Germany in 2022

We analyzed a total of 1,446,420 patients (10% sample of *N* = 14,464,195 cases) who were hospitalized in Germany in 2022 (Table 1). The average age of all inpatients was 63.16 years, 52.50% were female. 15.87% (*N* = 229,619) of inpatients had ASCVD, a majority of which featured coronary artery disease, 6.40% (*N* = 92,539) of inpatients were classified as obese, with 3.00% assigned to obesity grade I, 1.68% to grade II, 1.45% to grade III, and 0.27% referred to unspecified levels of obesity. 17.12% (*N* = 247,691) of inpatients had type 2 diabetes mellitus. Moreover, 43.35% presented with arterial hypertension and 4.87% with hypercholesterolemia. Symptomatic heart failure NYHA II-IV was diagnosed in 9.16% of cases.

**Table 1:** Characteristics of patients hospitalized in Germany in 2022

N	1,446,420	
Age in years, mean (SD)	63.16 (19.30)	
Female	759,323	52.50%
ASCVD	229,619	15.87%
CAD	149,078	10.31%
Previous MI	39,723	2.75%
Ischemic or hemorrhagic stroke	32,172	2.22%
Peripheral vascular disease	55,064	3.81%
Obesity	92,539	6.40%
Obesity grade I	43,346	3.00%
Obesity grade II	24,323	1.68%
Obesity grade III	20,972	1.45%
Unspecified level of obesity	3,898	0.27%
Type 2 diabetes mellitus	247,691	17.12%
Hypercholesterolemia	70,435	4.87%
NYHA II-IV	132,564	9.16%
Arterial hypertension	626,994	43.35%
Previous cardiac surgery	43,826	3.03%
Carotid disease	11,515	0.80%
COPD	90,445	6.25%
Pulmonary hypertension	25,221	1.74%
Renal disease, GFR < 30ml/min or < 15ml/min	45,615	3.15%
Atrial fibrillation	206,594	14.28%

*ASCVD* atherosclerotic cardiovascular disease, *CAD* coronary artery disease, *COPD* chronic obstructive pulmonary disease, *GFR* glomerular filtration rate, *MI* myocardial infarction, *N* number of patients, *NYHA* New York Heart Association, *SD* standard deviation

### Proportion of patients suffering from ASCVD, type 2 diabetes mellitus, obesity or a combination thereof

**Fig. 1:**
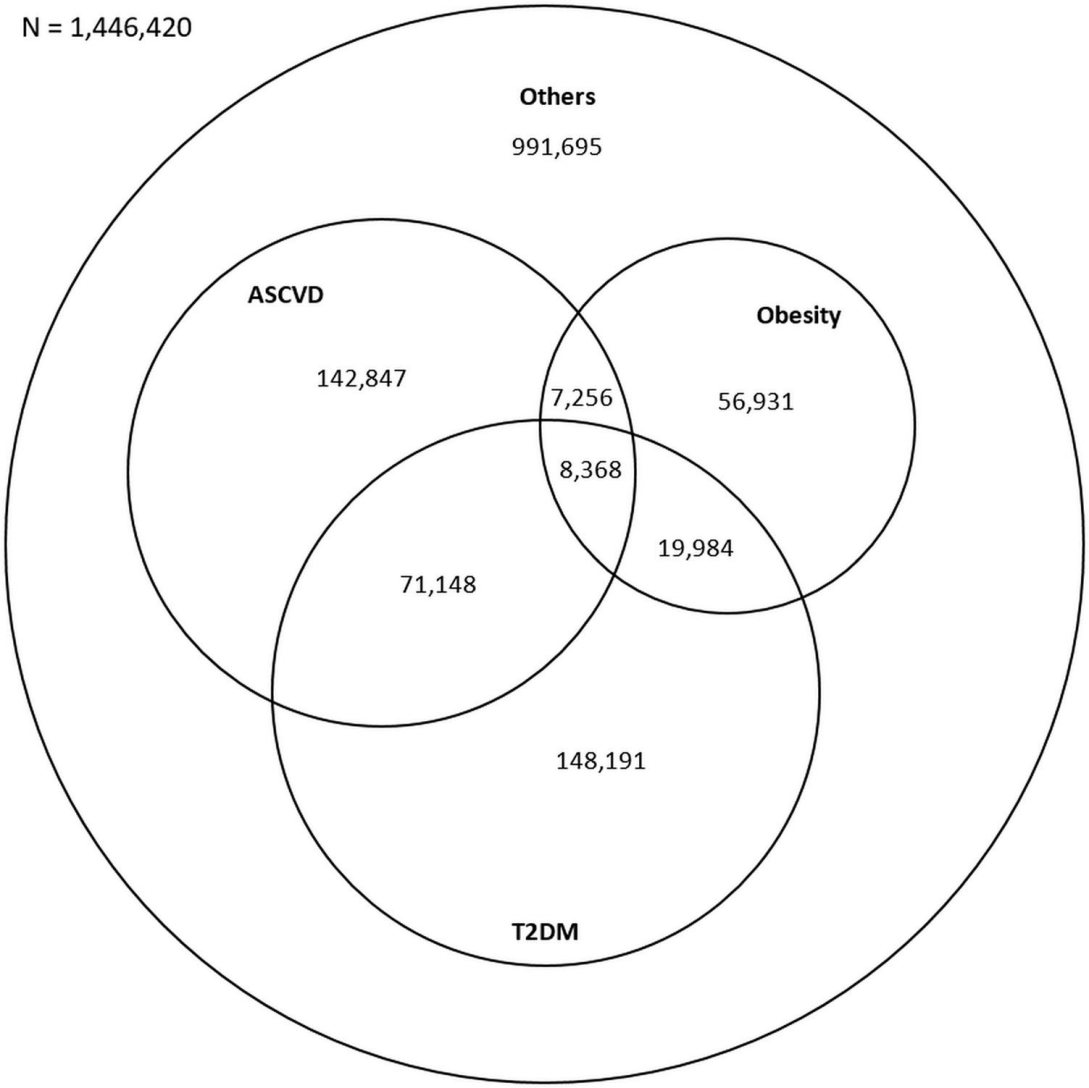
Proportion of patients suffering from ASCVD, type 2 diabetes mellitus, obesity or a combination thereof. ASCVD: atherosclerotic cardiovascular disease; N: number of patients; T2DM: type 2 diabetes mellitus

Of the 229,619 patients with ASCVD, 15,624 patients had concomitant obesity, 79,516 patients had concomitant type 2 diabetes mellitus (34.63% of ASCVD patients), and 8,368 featured all three disease entities combined (Fig. 1, Table 2). On the other hand, 142,847 patients had ASCVD without type 2 diabetes mellitus or obesity. The diagnosis of obesity was assigned to *N* = 92,539 of all inpatients (6.40%), of which 28,352 inpatients (30.64% of obese patients) had concomitant type 2 diabetes mellitus. Conversely, 28,352 out of 247,691 inpatients with type 2 diabetes mellitus were classified as obese. A majority of all inpatients (*N* = 991,695) presented with neither ASCVD nor diabetes mellitus nor obesity.

**Table 2:** Characteristics of patients with ASCVD, type 2 diabetes mellitus, and obesity

	ASCVD		T2DM		Obesity	
N	229,619		247,691		92,539	
Age in years, mean (SD)	73.95 (11.34)		73.19 (11.91)		61.12 (15.78)	
Female	78,821	34.33%	108,007	43.61%	49,073	53.03%
ASCVD	229,619	100.00%	79,516	32.10%	15,624	16.88%
CAD	149,078	64.92%	52,698	21.28%	10,719	11.58%
Previous MI	39,723	17.30%	14,218	5.74%	3,375	3.65%
Ischemic or hemorrhagic stroke	32,172	14.01%	8,552	3.45%	1,318	1.42%
Peripheral vascular disease	55,064	23.98%	23,701	9.57%	3,949	4.27%
Obesity	15,624	6.80%	28,352	11.45%	92,539	100.00%
Obesity grade I	8,381	3.65%	11,640	4.70%	43,346	46.84%
Obesity grade II	4,031	1.76%	7,833	3.16%	24,323	26.28%
Obesity grade III	2,371	1.03%	7,384	2.98%	20,972	22.66%
Unspecified level of obesity	841	0.37%	1,495	0.60%	3,898	4.21%
Type 2 diabetes mellitus	79,516	34.63%	247,691	100.00%	28,352	30.64%
Hypercholesterolemia	28,507	12.41%	21,075	8.51%	5,854	6.33%
NYHA II-IV	56,079	24.42%	46,715	18.86%	11,925	12.89%
Arterial hypertension	149,346	65.04%	167,287	67.54%	52,603	56.84%
Previous cardiac surgery	26,857	11.70%	16,101	6.50%	2,972	3.21%
Carotid disease	5,985	2.61%	3,644	1.47%	739	0.80%
COPD	25,994	11.32%	24,985	10.09%	8,150	8.81%
Pulmonary hypertension	9,234	4.02%	8,083	3.26%	2,338	2.53%
Renal disease, GFR < 30ml/min or < 15ml/min	16,431	7.16%	19,315	7.80%	3,241	3.50%
Atrial fibrillation	67,194	29.26%	60,739	24.52%	14,496	15.66%

*ASCVD* atherosclerotic cardiovascular disease, *CAD* coronary artery disease, *COPD* chronic obstructive pulmonary disease, *GFR* glomerular filtration rate, *MI* myocardial infarction, *N* number of patients, *NYHA* New York Heart Association, *SD* standard deviation

### Characteristics of patients suffering from ASCVD and obesity without diabetes mellitus

7,256 patients had ASCVD and obesity, but no diabetes mellitus, corresponding to 0.50% of all inpatients or 3.16% of all ASCVD patients. The mean age of that subpopulation was 68.81 years of whom 33.39% were female (Table 3). In comparison, the proportion of women among all patients with ASCVD was similar (34.33%, *p* = 0.099). However, it was significantly lower than in patients with type 2 diabetes mellitus (43.61%, *p* < 0.001) and with obesity (53.03%, *p* < 0.001). The mean age of all patients with ASCVD was 73.95 years, with type 2 diabetes mellitus 73.19 years, and with obesity 61.12 years.

**Table 3:** Characteristics of patients suffering from ASCVD and obesity without diabetes mellitus

N	7,256	
Age in years, mean (SD)	68.81 (11.59)	
Female	2,423	33.39%
ASCVD	7,256	100.00%
CAD	5,041	69.47%
Previous MI	1,655	22.81%
Ischemic or hemorrhagic stroke	680	9.37%
Peripheral vascular disease	1,404	19.35%
Obesity	7,256	100.00%
Obesity grade I	4,292	59.15%
Obesity grade II	1,708	23.54%
Obesity grade III	873	12.03%
Unspecified level of obesity	383	5.28%
Type 2 diabetes mellitus	0	0.00%
Hypercholesterolemia	1,071	14.76%
NYHA II-IV	1,950	26.87%
Arterial hypertension	4,883	67.30%
Previous cardiac surgery	763	10.52%
Carotid disease	177	2.44%
COPD	949	13.08%
Pulmonary hypertension	321	4.42%
Renal disease, GFR < 30ml/min or < 15ml/min	339	4.67%
Atrial fibrillation	2,033	28.02%

*ASCVD* atherosclerotic cardiovascular disease, *CAD* coronary artery disease; *COPD* chronic obstructive pulmonary disease, *GFR* glomerular filtration rate, *MI* myocardial infarction, *N* number of patients, *NYHA* New York Heart Association, *SD* standard deviation

69.47% of ASCVD inpatients with obesity but without diabetes mellitus had a coronary artery disease and 22.81% a history of myocardial infarction, both of which was significantly higher than among all patients with ASCVD (*p* < 0.001). 9.37% had a history of ischemic or hemorrhagic stroke and 19.35% peripheral vascular disease, both of which was significantly lower than among all patients with ASCVD (*p* < 0.001). A majority were assigned to grade I obesity (59.15%), 23.54% were classified as grade II, and 12.03% as grade III obesity. In 5.28% of cases, the level of obesity was not specified. Compared to all inpatients with ASCVD, obese ASCVD patients without diabetes mellitus featured significantly more hypercholesterolemia (14.76%), symptomatic heart failure NYHA II-IV (26.87%), arterial hypertension (67.30%), as well as COPD (13.08%; all *p* < 0.001). However, there were lower rates of previous cardiac surgery (10.52%; *p* = 0.002), renal disease (4.67%; *p* < 0.001), and atrial fibrillation (28.02%; *p* = 0.022).

## Discussion

In total, the present study examined 1,446,420 patients who were hospitalized in Germany in 2022 using a random 10% sample. Of these, 229,619 had been diagnosed with ASCVD, 92,539 with obesity, and 247,691 with type 2 diabetes mellitus. An overlap of ASCVD with diabetes mellitus or obesity was noted in 86,772 ASCVD patients, corresponding to 37.8% of all ASCVD patients. These 37.8% of all ASCVD inpatients may qualify for GLP-1 RA therapy. They had more hypercholesterolemia, heart failure NYHA II-IV, arterial hypertension, and COPD.

The sex ratio among all inpatients was approximately 50:50, whereas among all patients with ASCVD as well as those with ASCVD and obesity without diabetes mellitus only around 1/3 were women. These ratios of females are similar to those included in the SELECT trial (27.7%) [27]. In addition, patients with ASCVD as well as ASCVD and obesity without diabetes mellitus were older with a mean age of 73.9 and 68.8 years, respectively, than the overall patient cohort at 63.2 years. Likewise, in a previous study [30], we saw that among patients with coronary artery disease who underwent coronary angiography, the mean age was 69.8 years and 29.3% were women.

A Germany-wide survey study [31], which was conducted by telephone among 62,214 citizens, investigated the prevalence of CVD, defined as history of myocardial infarction, coronary heart disease, stroke, as well as congestive heart failure. CVD prevalence was between 10.0 and 15.8% depending on the federal state, which appears only slightly lower than the ASCVD prevalence amongst our inpatient cohort (15.9%).

Stein et al. [32] also analyzed the prevalence of obesity among 204,751 adult individuals in Germany. In that cohort, 19.9% of adults were classified as obese. Obesity is attributed a global prevalence of 14.0–18.5% [7]. In our dataset of inpatients, however, the prevalence of obese patients was as low as 6.4%, of which 30.6% also had diabetes mellitus.

In contrast, the diagnosis of type 2 diabetes melllitus was made in 17.1% of inpatients in our study, whereas the prevalence in Germany after standardization was 7.1% in 2010 [33]. Diabetes mellitus was attributed a global prevalence of 10.5% [6] in 2021. Auzanneau et al. [34] describe a rate of 17.1% for inpatients in Germany in 2017, which corresponds to ours. The authors explain the substantially higher rate of type 2 diabetes mellitus among inpatients by a possibly higher need for inpatient stays among older patients and those suffering from diabetes mellitus. However, the relative increase in type 2 diabetes mellitus between 2015 and 2040 in Germany is estimated in the literature to be between 54 and 77% [35].

The prevalence of obesity we observed is lower than the presumed prevalence in the population. However, the prevalence of diabetes mellitus is probably overestimated in our study. This may affect a possible transferability to the outpatient population. Due to the available data, we were only able to include patients with a BMI of 30kg/m^2^ or higher. The fact that those with a BMI of 27kg/m^2^ or higher according to the SELECT trial [27] could not be considered may also have had an impact. However, there are patients with both diabetes mellitus and overweight or obesity. It is therefore not clear whether and, if so, to what extent this might have influenced the eligible cohort. When interpreting the data, possible contraindications must also be taken into account, which were not explicitly addressed for reasons of clarity of this study. However, since it can be assumed that noticeably more people will benefit, our data provide a good basis for further research and considerations, as they most likely represent a minimum of the estimated eligibility for GLP-1 RA therapy.

Our investigation demonstrates that a relevant number of inpatients with ASCVD may qualify for GLP-1 RA therapy. Therefore, integrating an automated alignment of coded diagnoses in health records may be a potential option to encourage consideration of GLP-1 RA therapy in the future.

The strength of our study is the investigation of a large sample representative of more than 14 million inpatients in Germany. However, in administrative data, coding errors may occur and comorbidities often not relevant to reimbursement, such as obesity, are possibly underreported. Also readmissions of a patient cannot be excluded. Due to the available data, there is no information on the exact BMI of the patients. In addition, our study only comprises inpatient data and information on obesity with a BMI of 30kg/m^2^ or higher [36, 37]. In the SELECT trial [27], however, ASCVD patients with overweight or obesity were already included with a BMI of 27kg/m^2^ or higher.

## Conclusions

Based on our analysis of inpatients in Germany, more than one-third of patients with ASCVD may qualify for GLP-1 RA therapy due to concomitant diabetes mellitus or obesity. That corresponds to about 6% of the patients hospitalized in Germany.

## Data Availability

Data are available upon reasonable request. The patient data are stored on the server of the Federal Statistical Office of Germany and are not available due to data protection. The calculated raw data are sent anonymized to the scientist.
